# Cannabigerol at the Interface of the Gut Microbiota and EndoCannabinoidome: Mechanistic Insights into Inflammation and Pain Modulation

**DOI:** 10.3390/medsci14050560

**Published:** 2026-09-10

**Authors:** Gloria Marisol Castañeda-Ruelas, Lucía Elhy Grijalva-Contreras, Geovanna Nallely Quiñonez-Bastidas

**Affiliations:** 1 Faculty of Chemical and Biological Sciences, Autonomous University of Sinaloa, Culiacan 80010, Sinaloa, Mexico; gloria.ruelas@uas.edu.mx; 2 General and Community Medicine Program, Hermosillo Academic Unit, Sonora State University, Hermosillo 83100, Sonora, Mexico; lucia.grijalva@ues.mx; 3 Neuropharmacology and Pain Laboratory, Department of Research, Center for Research and Teaching in Health Sciences, Autonomous University of Sinaloa, Culiacan 80030, Sinaloa, Mexico

**Keywords:** cannabigerol, enteric endocannabinoid system, enteric nervous system, gut microbiota, inflammation, pain

## Abstract

Recent years have seen growing medical interest in the influence of crosstalk between the gut microbiota and the endocannabinoidome (eCBome) on inflammation and pain modulation. Growing evidence indicates that gut microbiota can modify the effects of several marketed drugs, including analgesics. Cannabigerol (CBG) is an overlooked phytocannabinoid with a broad pharmacological spectrum and no psychotropic effects, which has anti-inflammatory and antinociceptive properties. This review aims to provide a comprehensive exploration of CBG and its role at the intersection of gut microbiota and eCBome, detailing the pharmacological mechanisms by which it acts as a promising therapeutic agent to modulate chronic inflammation and pain. Our data review suggests that CBG could act on the eCBome by activating CB2, PPARs, TRPV1, TRPA1, and α2-adrenergic receptors, while suppressing cellular and molecular mechanisms of inflammation, such as TNFα, COX-2, iNOS, IL-1β, and IL-6, and increasing antioxidant factors. These receptors and enzymes are distributed across neurons, glial, immune, and epithelial cells, which can also positively modulate gut microbiota and its metabolites, producing neurotransmitters, cytokines, and enzymes that regulate eCBome tone, and generating cannabinoid-mimetic compounds as part of pleiotropic functions. Nevertheless, CBG may exert direct effects on gut microbiota, promoting eubiosis and symbiotic bacteria. Moreover, there are no specific preclinical assays that demonstrate how CBG modulates the bidirectional communication between the gut microbiota and eCBome, addressing the specific mechanism involved in eCBome activation, and determining whether its anti-inflammatory and analgesic effects are dependent on gut microbiota type. Therefore, studies are required to evaluate this hypothesis to achieve translational medicine impact.

## 1. Introduction

Pain is characterized as an unpleasant sensory and emotional experience that is associated with, or resembles, the sensation of actual or potential tissue damage [1]. Pain and inflammation can be intrinsically related; inflammation in the tissue leads to the development of pain, particularly inflammatory pain, which is one of the most common types of chronic pain. Local, peripheral, and central inflammatory responses in the body are related to acute and chronic pain [2].

Moreover, pain is also one of the five signs of an inflammatory process. When inflammation is not resolved, it turns into chronic pain. This latter has a severe impact on the quality of life of the patients who suffer from this condition. According to the epidemiology of pain, nearly 30% of the global population could be living with chronic pain [3].

Furthermore, neuropathic pain is caused by pathology or damage affecting the somatosensory nervous system, while nociplastic pain results from altered processing of pain signals despite no clear evidence of damage or pathology of the somatosensory system. It is well-known that both share an important inflammatory feedback loop, which increases pain sensitivity and promotes its maintenance [4].

Pain is always influenced by a series of social, psychological, and biological factors, as well as personal habits, which can modulate nociception and the perception of pain, contributing to the individualized experience of it [5]. Even though the predisposition of these factors is not completely known, understanding the role of those risk factors is needed for the best approach to pain management [6]. In this way, one of the novel biological factors proposed as a target for pain regulation is the gut microbiota [7].

The gut microbiota is a microenvironment in which several microorganisms live in the human body and play a pivotal role in host health. In recent years, the relationship between the gut microbiota and many diseases has been studied, especially pathological conditions such as chronic inflammation [8,9]. There is growing interest in the hypothesis that dysbiosis in the gut microbiota and its interactions with the enteric nervous system (ENS) and the central nervous system (CNS) may play a role in the control of behavior and the regulation of pain [10].

When the homeostasis of gut microbiota is lost, it can produce neuroinflammation through an inflammatory signaling cascade, composed of neurotransmitters, short-chain fatty acids (SCFAs), chemokines, and cytokines that act on the gut and ENS, including the vagus nerve, which transmits harmful stimuli to the CNS. In parallel, when these inflammatory substances are released into the circulation, they can produce peripheral (dorsal root ganglia [DRG] and trigeminal ganglia neurons [TG]) and central sensitization, neural plasticity, microglial activation, changes in neuronal excitability, and can influence the size and activity of supraspinal areas related to pain processing [10,11]. Furthermore, the nociceptor neurons in the peripheral nervous system (PNS) and CNS respond by releasing neurotransmitters and inflammatory mediators to provide feedback on the pain process [12].

At the beginning of this century, two important studies contributed to explaining the relationship between gut microbiota and pain. The analysis of the gut microbiota of patients with irritable bowel syndrome (IBS) was performed using gas chromatography and mass spectrometry, which revealed specific changes in the eubacterial species of the bowel in pathological conditions, demonstrating the role of dysbiosis in IBS [13]. Moreover, preclinical models demonstrated that increased hypernociceptive responses to inflammatory stimuli were observed in mice with naïve microbiota; meanwhile, germ-free mice had a decrease in nociception, which was reversed by the administration of feces from the mice with naïve microbiota [14].

It is well documented that the gut microbiota-brain axis is involved in the pathogenesis of some painful inflammatory chronic conditions, such as visceral chronic pain, IBS, and chronic pelvic pain, as well as migraine attacks [15,16]. In addition, the gut microbiota extends its regulation to mediate the analgesic effects of common drugs used to treat chronic pain [17]. Research strongly links the gut microbiota to the development of tolerance, dependence, and pain modulation of opioids like morphine [18]. Another relevant finding is the pivotal role of the eCBome in pain and the cross-talk regulation with the gut microbiota [19,20].

Regarding this, previous preclinical studies have demonstrated that non-psychotropic phytocannabinoids, such as cannabigerol (CBG), induce a strong anti-inflammatory effect, showing the capacity of such molecules to modulate the gut microbiota and neuroimmune regulation [21,22]. Currently, CBG has drawn interest because its effects are extended to modulate several molecular targets in the pain pathways in different models of pain [23,24]. Notwithstanding, no evidence indicates how the gut microbiota could influence cannabinoid-based analgesic responses.

In this descriptive review, experimental and clinical studies available in electronic databases (PubMed, Scopus, Google Scholar, etc.) were critically and meticulously analyzed. These studies were employed to describe the characteristics of CBG and elucidate the molecular mechanism it exerts on the gut microbiota-eCBome axis. Additionally, the implications of CBG on pain and inflammatory disorders were comprehensively explored. Therefore, this review aimed to provide a comprehensive exploration of CBG and its role at the intersection of the gut microbiota and the eCBome, detailing the pharmacological mechanisms through which it acts as a promising therapeutic agent to modulate chronic inflammation and pain.

## 2. The Gut Microbiota

The role of gut microbiota requires the definitions of microbiota and microbiome concepts. Microbiota is defined as a community of living microorganisms (bacteria, fungi, viruses, and others) that resides in a specific environment. The microbiome concept refers to the set of diverse microbial genomes, structural elements, metabolites, and environmental conditions [25], consolidating as the second genome of the host [26]. Therefore, the microbiota is an integral component of the microbiome, which performs the interactions that preserve the homeostasis of the host.

Particularly, the gut microbial community coexists as symbionts and mutualistic microorganisms with host cells, maintaining a direct interaction with the immune system [27]. The gut microbiota acts as a dynamic functional entity that actively regulates metabolism and pharmacological interactions (bioavailability and efficiency) with exogenous agents [26,28]. The main functions include the mediation of metabolic processes, immune maturation, and the reinforcement of the intestinal mucosa [29]. The primary contributions and functions of gut microbiota encompass the harvest of energy from dietary sources, the production of SCFAs, the detoxification of xenobiotics, the active defense against pathogens through competition or antagonism, and the preservation of mucosal barrier integrity [25,29,30,31].

The metabolites of gut microbiota can be classified into three main categories: (i) produced from the diet, such as SCFAs (acetate, propionate, & butyrate), tryptophan and indole derivatives, branched-chain amino acids (Leu, Ile, Val), gases (H_2_S, H_2_, CO_2_, CH_4_, NO), N-oxide-trietilamine, neurotransmitter (dopamine, catecholamine, 5-hydroxy tryptamine [5-HT], and gamma-aminobutyric acid [GABA]) and choline. (ii) Produced by the host, like secondary bile acids, and (iii) produced in novo as essential vitamins (B complex and K), structural bacterial components (lipopolysaccharides and peptidoglycans), and the antimicrobial proteins (bacteriocins) [32,33,34,35].

The functions of gut microbiota and the production of metabolites establish a biological framework to regulate immune and metabolic processes and determine visceral sensitivity and analgesic responses against inflammatory signals, placing these microbial metabolites as ligands of the gut–brain axis [36].

### 2.1. Composition of Gut Microbiota: Pro-Nociceptive and Anti-Nociceptive Bacteria

The healthy and dynamic balance in the gut ecosystem is called eubiosis [37]. The dynamic interaction between intrinsic factors (host genetics, age, birth method, immune system status) and extrinsic factors (diet, geographic environment, lifestyle) regulates the composition and balance of gut microbiota [36]. In contrast, the alteration of gut microbiota is named dysbiosis and implies qualitative and quantitative changes in microbial density, loss of beneficial functions, and proliferation of opportunistic pathogens [25]. Gut microbial dysregulation could be attributed to multiple factors, such as tobacco use, alcohol consumption, overweight, overuse of medications (especially antibiotics), exposure to environmental contaminants, and food additive intake [38].

The gut microbiota is characterized by its abundance and diversity, harboring >100 trillion microorganisms in the human gastrointestinal tract, functioning as a dynamic and fundamental system for human health [39,40]. Bacteria, yeast, and viruses compose the gut microbiota. Phylogenetic analysis indicates that the gut microbiota of a healthy host is dominated primarily by bacterial phyla such as *Firmicutes*, *Bacteroidetes*, *Actinomycetota*, *Verrucomicrobia*, *Proteobacteria*, and *Fusobacteria*. It should be noted that *Firmicutes* and *Bacteroidetes* represent 90% of the gut microbiota [29]. The phyla proportions can vary between hosts [41] but human health depends on the balance among these phyla, especially the *Firmicutes*/*Bacteroidetes* ratio [29].

Also, the gut microbiota plays a fundamental role in the etiology of diverse neurological pathologies, influencing the processing of different pain modalities (visceral, chronic, and inflammatory) and pathophysiologies like headache and neuropathic pain [7]. Although the current literature lacks a generalized classification of bacterial phyla as pro-nociceptive and anti-nociceptive bacteria, the functional consequences of certain bacterial species belonging to these phyla and alterations in their composition or proportion suggest a fundamental role for nociceptive and inflammatory signaling [42].

**Figure 1:**
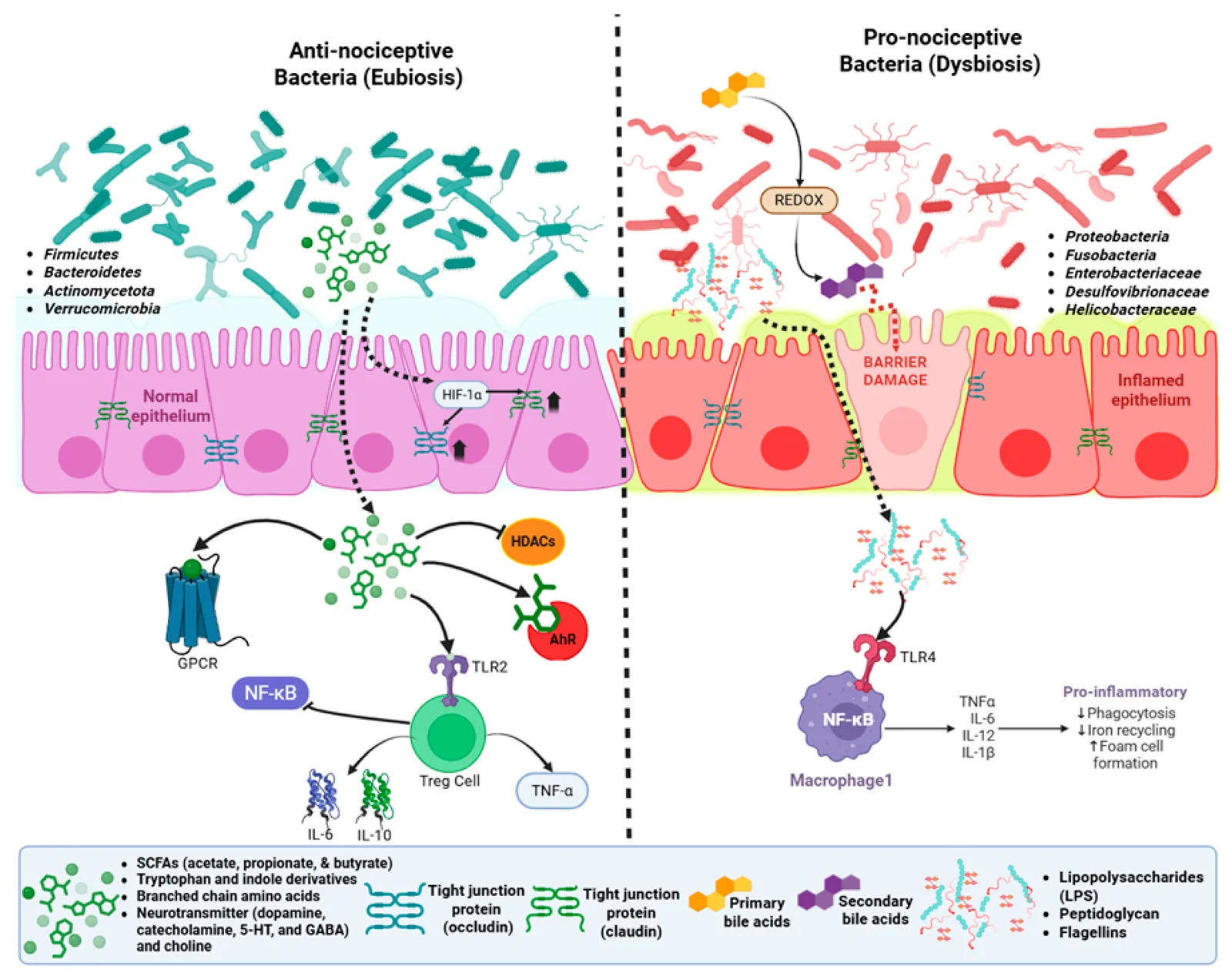
Functional classification and biological effects of gut microbiota in nociceptive modulation. Comparative scheme of the gut microbiota regulating the pain threshold and the host’s inflammatory tone. The left panel illustrates the role that antinociceptive bacteria (green) would play by producing homeostatic metabolites (SCFAs and neurotransmitters) that potentially activate antinociceptive and anti-inflammatory pathways and preserve the epithelial barrier. The right panel illustrates the role of pro-nociceptive bacteria (red) in eliciting enteric neuroinflammation, hypersensitivity, and mucosal damage through the production of structural components such as lipopolysaccharides (LPS), peptidoglycan, and flagellins. Arrows indicate activation of cells, receptors, and cytokines; dashed arrows indicate the effects through the epithelial barrier, and blunt arrows indicate inhibitory effects.

In this context and for this review, certain bacteria can be classified into two groups based on their role in pain and inflammatory signaling: pro-nociceptive and anti-nociceptive bacteria (Figure 1). The dual function of this interaction defines the neuro-immunological balance of the host, influencing sensitivity and pain threshold. In particular, the abundance of symbionts may play a potential role as anti-nociceptive bacteria, while the proliferation of pathobionts may act as pro-nociceptive bacteria.

The anti-nociceptive bacteria are mainly represented by certain bacterial species belonging to the *Firmicutes*, *Actinomycetota*, and *Verrucomicrobia* phyla [29]. These bacteria are symbionts with the host, utilizing various metabolic pathways to produce several metabolites that serve as energy sources and signaling essential molecules for maintaining the integrity of the mucosal barrier, modulating the immune system, and providing analgesic effects to diverse pain modalities [38,43,44].

Regarding this, *Firmicutes* (>200 genera of *Lactobacillus*, *Bacillus*, *Clostridium*, *Enterococcus*, and *Ruminococcus*) ferment complex carbohydrates. Meanwhile, *Bacteroidota* (like *Bacteroides* and *Prevotella*) degrade carbohydrates derived from the diet or from the host, whose products are key in energetic metabolism and immune homeostasis. Moreover, *Actinomycetota* (like *Bifidobacterium*) regulate innate immunity, and *Verrucomicrobiota* (*Akkermansia* spp.) preserve the integrity of intestinal mucus and prevent systemic inflammation [29].

The pro-nociceptive bacteria predominantly belong to the phyla *Proteobacteria* and *Fusobacteria*. However, this classification can change based on individual health and dietary factors. These bacteria can perform redox reactions and amino acid biosynthesis. Nevertheless, their excessive proliferation is closely associated with inflammatory and pathological processes [29,38]. The bacteria belong to *Enterobacteriaceae* (*Escherichia*, *Shigella*), *Desulfovibrionaceae* (*Desulfovibrio*), *Helicobacteraceae* (*Helicobacter*), and *Fusobacteriaceae* (*Fusobacterium*) families, which are highlighted as the main pathobionts [29].

### 2.2. Microbiota Gut–Brain Axis

The gastrointestinal tract possesses a complex network of receptors (vanilloid—TRPV, proteases, cholecystokinin, serotonin, and cannabinoids) and ionic channels (ATP, Na^+^/Ca^2+^) that sense pain, which could be activated by microbial metabolites and changes generated by microbiota in the intestinal environment [45]. The main pathways that facilitate the bidirectional communication of the microbiota gut–brain axis are the neuronal pathway (vagus nerve and ESN), the endocrine-metabolic pathway, and the immune pathway (cytokines) [33]. This axis transmits signals and returns responses to modify the vital functions of the gut, such as motility, permeability, immune response, acidity, and mucus production [46].

The neuronal pathway is the main communication between the gut and brain and is formed by the ENS and vagus nerve [47]. The ENS (known as the second brain) is a massive network of 200–600 million neurons distributed on the gastrointestinal tract, which controls essential functions and processes chemical signals in an autonomous form [48]. The vagus nerve is the primary component of the parasympathetic system and the connection between the gut system and the CNS [49]. It functions as a sensor of microbial and inflammatory signals via afferent fibers and acts as a modulator of immune response through efferent fibers. The efferent fiber mediates the cholinergic inflammatory pathway, being able to attenuate peripheral inflammation and reduce intestinal permeability [50].

The metabolic-endocrine pathway is based on the release of microbial metabolites (mainly butyrate) into the bloodstream. These metabolites act on endocrine receptors (such as G protein-coupled receptors [GPCR]) that modulate central homeostasis, protect the nervous system [51], and regulate immune system functions. As a neuroinflammatory mediator, it preserves homeostasis through immunological tolerance or, in a state of dysbiosis, promotes neuroinflammation and neuronal damage [42].

According to the functions and molecular mechanisms of bacteria (Figure 1), at the molecular level, the metabolites produced by anti-nociceptive bacteria act as inhibitors of histone deacetylases (HDACs) and as ligands for receptors, such as G-protein-coupled receptors (GPR41, GPR43, and GPR109A), Toll-like receptor 2 (TLR2), and the aryl hydrocarbon receptor (AhR) [43,52]. This molecular interaction promotes the tolerogenic phenotype and the induction of regulatory T lymphocytes (Tregs) for the synthesis of anti-inflammatory such as interleukin-10 (IL-10), the suppression of nuclear factor kappa B (NF-kB), and the release of pro-inflammatory mediators, tumor necrosis factor-alpha (TNF-α) and interleukin-6 (IL-6) [44]. At the structural level, the hypoxia-inducible factor (HIF-1α) is stabilized, strengthening the tight junction proteins (claudins and occludins) and preserving the mucosal barrier integrity, avoiding the translocation of pro-nociceptive antigens to the bloodstream [53].

The molecular mechanism by which pro-inflammatory bacteria induce pain and inflammatory disorders is related to the production of secondary bile acids, which compromise the integrity and permeability of epithelial barriers (Figure 1). This process allows the translocation of bacterial components like lipopolysaccharides (LPS), peptidoglycan, and flagellins to the bloodstream [42]. These antigens are ligands of the Toll-like receptor 4 (TLR4) presented in pro-inflammatory macrophages, triggering an intracellular signaling cascade by NF-kB, mitogen-activated protein kinase (MAPK), and releasing pro-inflammatory cytokines TNF-α, interleukin 1 beta (IL-1ß), IL-6, and interleukin 12 (IL-12) [38,43,52]. This chronic inflammatory state and the dysfunctional immune response facilitate the development of severe pathologies like inflammatory intestinal disease, metabolic disorders (diabetes and obesity), and systemic autoimmune diseases [36,42].

## 3. The Endocannabinoid System in the Gastrointestinal Tract

The eCBome plays a fundamental role in the neuroimmune axis and comprises a complex network of receptors, ligands, and enzymes distributed throughout the body [54]. Initially, its importance was discovered in the PNS and CNS; however, its signaling is not limited to the brain but extends to other parts of the body, such as the gastrointestinal tract [55,56,57].

### 3.1. Biotransformation of eCBome: Ligands, Receptors, and Enzymes

The typical canonical endocannabinoids are anandamide (AEA) and 2-arachidonoylglycerol (2-AG), which interact with the cannabinoid receptor type 1 (CB1) (primarily CNS) and cannabinoid receptor type 2 (CB2) (primarily peripheral and immune systems) [58,59,60]. For the synthesis of endocannabinoids, there are two major enzymes implicated: diacylglycerol lipase (DAGL) for 2-AG and *N*-acylphosphatidylethanolamine phospholipase D (NAPE-PLD) for AEA. Post-production, 2-AG hydrolysis may be mediated by monoacylglycerol lipase (MAGL), αβ hydrolase 12 (ABHD12), or αβ hydrolase 6 (ABHD6). Meanwhile, AEA can be hydrolyzed by fatty acid amide hydrolase (FAAH) or *N*-acylethanolamine acid amidase (NAAA) [61,62].

**Figure 2:**
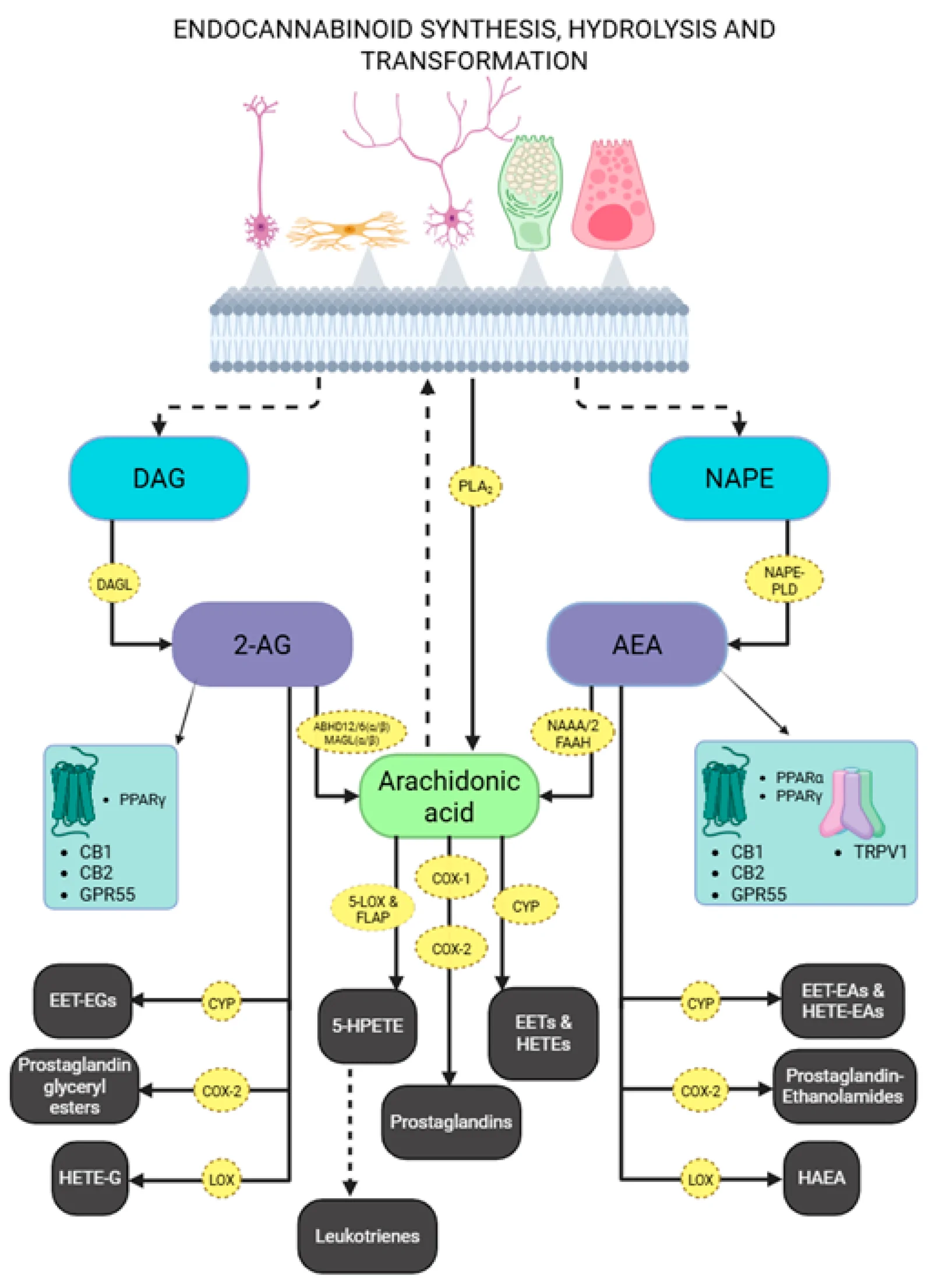
Molecular machinery and biotransformation pathways of the endocannabinoidome in the gastrointestinal tract. Qualitative schematic representation of the metabolic balance of the canonical ligands (purple color): anandamide (AEA) y 2-araquidonilglicerol (2-AG) produced from membrane phospholipids (blue color) as diacylglycerol (DAG) and N-acylphosphatidylethanolamide (NAPE). CBG synthesis occurs through the enzymes N-acylphosphatidylethanolamine phospholipase D (NAPE-PLD) and diacylglycerol lipase (DAGL), while the primary mechanisms of enzymatic degradation are mediated by fatty acid amide hy-drolase (FAAH), N-acylethanolamine acid amidase (NAAA), monoacylglycerol lipase (MAGL), αβ hydrolase 12 (ABHD12), or αβ hydrolase 6 (ABHD6). Alternative metabolic pathways are mediated by cyclooxygenase-2 (COX-2), lipoxygenase (LOX), or epoxygenase/cytochrome P450 (EPOX/CYP450), which divert arachidonic acid towards endocannabinoids and the production of lipid mediators and hydroperoxides (gray color) involved in the modulation of the inflammatory tone and visceral pain signaling. All enzymes are indicated in yellow ovals. The main targets of endocannabinoids are grouped in the clear cyan square. Arrows indicate enzymatic-mediated processes, while dashed arrows indicate non-enzymatic-mediated processes. Small arrows indicate the targets (receptors) of endocannabinoids.

In the same way, a process of transformation can occur through the enzyme cyclooxygenase-2 (COX-2), lipoxygenase (LOX), or epoxygenase/cytochrome P450 (EPOX/CYP450) (Figure 2) [61,62]. COX-2 is an inducible enzyme expressed in inflammatory cells and tissues, and is one of the three major inflammation pathways, as well as EPOX/CYP450 and LOX. Together, they play a pivotal role in the production of prostaglandins, leukotrienes, and hydroxyeicosatetraenoic acids, which are involved in inflammatory and pain processes [63,64]. Hence, non-steroidal anti-inflammatory drugs (NSAIDs) and selective COX-2 inhibitors can extend their effects by increasing endocannabinoids, avoiding their biotransformation into inflammatory compounds [65].

On the other hand, the eCBome has machinery able to synthesize other cannabinoid-mimetic compounds, which are not properly the canonical ligands but imitate the structure or function of canonical endocannabinoids to interact with the cannabinoid receptors CB1 or CB2 [54]. Some examples of non-classical endocannabinoids found in the intestine are 2-oleoyglycerol (2-OG); 2-palmitoyglycerol (2-PG), oleoylethanolamide (OEA); *N*-docosahexaenoyl-ethanolamine (DHEA); *N*-linoleoyl-ethanolamine (LEA); 2-linoleoyl-glycerol (2-LG); *N*-Palmitoylenthanolamide (PEA), *N*-Stearoylethanolamide (SEA); *N*-arachidonoyl dopamine (NADA); 2-arachidonyl glyceryl ether (2-AGE or noladin ether) and O-arachidonoyl-ethanolamine (virodhamine) [66,67].

Endocannabinoids are also synthesized locally “on demand” when cells are activated. Since their lipophilic nature results in short-lived signaling, they are not limited to acting on typical receptors; endocannabinoid and endocannabinoid-mimetic compounds can act on several molecular targets [68].

One of the most significant findings occurred in the 2000s with the discovery of AEA as a full activator of the transient receptor potential cation channel subfamily V member 1 (TRPV1) [69]; subsequently, findings have confirmed the existence of other molecular targets, such as peroxisome proliferator-activated receptor α (PPARα); peroxisome proliferator-activated receptor γ (PPARγ), G protein-coupled receptor 55 (GPR55), G protein-coupled receptor 119 (GPR119), and other receptors which are potentially activated by endocannabinoids and endocannabinoid-mimetic compounds [56,67].

Stemming from their properties, endocannabinoids can regulate the gut microbiota through a mix of mechanisms described below, which focus on barrier integrity, immune tone, and neuronal and metabolic signals [70]. Notwithstanding, endocannabinoids can also mediate direct effects on the growth, physiology, and function of bacteria [71].

### 3.2. Signaling Pathways

**Figure 3:**
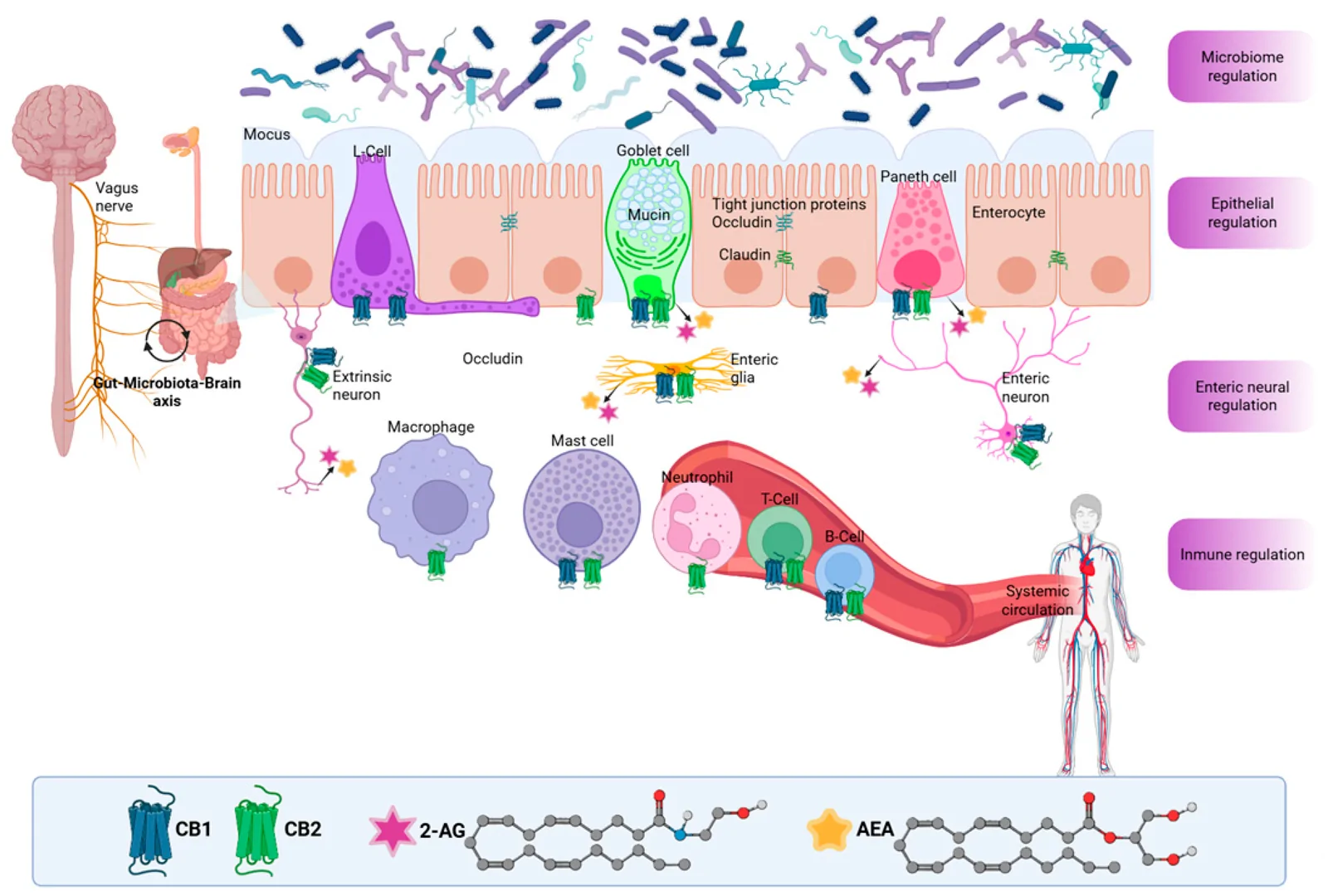
Cell signaling pathways and biological feedback are regulated through AEA (anandamide) and 2-AG (2-arachidonoylglycerol) in the gut–brain axis. Qualitative representation of the bidirectional communication between the mainly endogenous endocannabinoids and gut microbiota. The diagram illustrates the modulation of the immune response and mucosal barrier by qualitative signaling variations in AEA and 2-AG in response to nociceptive stimuli. The interaction of cannabinoid receptor type 1 (CB1) and cannabinoid receptor type 2 (CB2) regulates the phenotypic transition of macrophages and controls the visceral hypersensitivity induced by pathobionts. The enteric endocannabinoid system acts on enteric neural regulation through glial cells, enteric and extrinsic neurons that express CB1 and CB2 receptors. Similarly, certain specialized cells of the epithelial barrier express receptors for cannabinoids, enabling the preservation of barrier functions and preventing the permeability of pathogenic bacteria and their metabolites or neurotransmitters. Finally, enteric endocannabinoids can modulate the polarization of the gut microbiota from pathogenic to beneficial bacteria. The circular arrows represent a crosstalk between the gut microbiota and brain axis. The main biological effects of the endocannabinoids in the gut are highlighted in purple squares.

In the gut, the production of endocannabinoids occurs mainly in enteric neurons and subpopulations of extrinsic neurons, although they can also be produced in goblet cells, a specialized mucus-secreting epithelial cell, and Paneth cells, a crucial mediator of immunity and homeostatic processes (Figure 3) [72]. Once produced, endocannabinoids act on enteric glial cells (EGCs), immune-regulating cells such as mast cells, lymphocytes, neutrophils, and macrophages, which express the CB2 receptor [73,74,75]. Moreover, they can activate CB1/CB2 receptors to regulate enteric neurons, as well as epithelial cells and specific subtypes, goblet and Paneth cells, which mainly express CB2 receptors [76]. The activation of the CB2 receptor has been linked to diminished macrophage and plasma cell infiltration [73,77]. Also, EGCs express MAGL; hence, these cells act as regulators of the tone of 2-AG, which targets the CB2 receptor to relieve inflammation. Consequently, the eCBome regulates the interplay between neuroglial signaling to control motility and visceral pain [78,79,80,81].

The receptors CB1 and CB2 play a role in mast cells, controlling the activation and degranulation of these cells and subsequently avoiding the production of histamine, pro-inflammatory cytokines, and other inflammatory products [74,82,83]. The evidence has unveiled how the stimulation of the eCBome by AEA promotes Treg proliferation and IL-10 production, exerting anti-inflammatory and homeostatic effects on the gut [75].

Moreover, in the murine model, knockout of GPR55 has shown slightly but significantly lower inflammation in the gut compared with wild-type animals, highlighting the pro-inflammatory role of this receptor in gastrointestinal inflammation [84]. The antagonism of GPR55 in colitis models reduces inflammation through leukocyte reduction, especially macrophages, and secretion of pro-inflammatory cytokines [85]. Meanwhile, PPARα/γ receptors, two nuclear receptors, have been observed in immune and epithelial cells of the gut; both receptors are key in the signaling of cannabinoids in the gut [86,87].

PPARα activation is linked to maintenance of the intestinal barrier and to the development of tolerance toward the gut microbiota. This involves suppression of T helper cells (Th1/Th17) responses, regulation of the gut microbiota through upregulation of interleukin 22 (IL-22), and production of antimicrobial peptides, regenerating islet-derived protein 3 beta (RegIIIβ), regenerating islet-derived protein 3 gamma (RegIIIγ), and calprotectin [87,88]. Likewise, the anti-inflammatory effects of PPARα can be targeted by the inhibition of nitric oxide synthase and the consequent production of nitric oxide [89], which is important in the maintenance of microbiota homeostasis. Similarly, PPARγ receptor activation exerts an anti-inflammatory effect by inhibiting inflammatory cytokines, such as TNF-α, IL-6, and IL-1β [86]. In the same way, the activation of both nuclear receptors (PPARα/γ) produced a fast suppression of inflammation and pain in several models of inflammatory and neuropathic pain [90,91]. PPARα is also expressed in EGCs, highlighting how the modulation of these cells through cannabinoids maintains control in some inflammatory diseases [92].

Regarding this, the cross-talk regulation of PPARγ has been observed in the secretion [93] of certain bacterial metabolites that activate its receptors and promote an anti-inflammatory response [94]. Regarding this, microbiota-derived metabolites, such as OEA, activate the GPR119 receptor [95], which is expressed in vagal neurons and intestinal L-cells [93,96]. The activation of GPR119 has been related to the suppression of allodynic behavior in mice; perhaps GPR119 is linked to the stimulatory G protein alpha subunit (Gαs) and increased levels of cyclic adenosine monophosphate (cAMP) and consequent pain. Another signaling pathway of this receptor can be dependent on the inhibitory G protein alpha subunit (Gαi), and the Gq protein alpha subunit (Gαq), as well as independent pathways such as β-arrestin, which in turn can be responsible for the shift in signaling of GPR119 to induce relief of pain [97].

Finally, TRPV1 is a key player in pain perception and inflammation [98]. Its desensitization is linked to antinociception, and some therapeutic strategies have targeted the TRPV1 receptor, improving the clinical symptoms of pain [99,100,101].

In the gut, extrinsic TRPV1 neurons contribute to visceral hypersensitivity in an experimental colitis model [102], and this receptor is also expressed in mechanosensitive gastrointestinal vagal afferents [103]. TRPV1 nerve fibers have several functions in the gut, including motility, secretion, circulation, and visceral nociception. It has a large distribution in peptidergic and nitrergic neurons localized at the mucosa, submucosal layer, deep muscular plexus, circular muscle, myenteric plexus, and longitudinal muscle layer in the mouse rectal and colonic ENS [102]. In fact, TRPV1 has been proposed as a therapeutic target to relieve the pain in IBS, since it can reduce motility and pain in experimental approaches [104].

## 4. The Overlooked Non-Intoxicating Phytocannabinoid Cannabigerol

CBG is a non-intoxicating phytocannabinoid of *Cannabis sativa* L. that has historically received less attention than Δ9-tetrahydrocannabinol (THC) and cannabidiol (CBD), despite its central role in cannabinoid biosynthesis and its increasingly recognized pharmacological profile. In the 1960s, Mechoulam and Gaoni conducted the initial chemical characterization of cannabinoid acids, specifically cannabigerolic acid (CBGA). This work laid the basis for subsequent research on both neutral and acidic cannabinoids. In botanical terms, CBG is usually considered a minor cannabinoid because most cannabis chemotypes accumulate only low concentrations of its neutral form. However, this classification may underestimate its biological relevance, since CBGA is the biosynthetic precursor to the major acidic cannabinoids tetrahydrocannabinolic acid (THCA), cannabidiolic acid (CBDA), and cannabichromenic acid (CBCA) [105].

CBG has attracted renewed interest because it lacks the typical CB1-mediated intoxicating profile associated with THC, while interacting with several molecular targets involved in nociception, inflammation, gastrointestinal physiology, and neuroimmune regulation [106,107,108,109]. However, the pharmacological relevance of each target varies depending on the concentration, experimental model, formulation, and whether purified CBG or CBG-rich extracts are being evaluated. This distinction is important in inflammatory pain and gastrointestinal disorders, where neuronal excitability, immune activation, epithelial barrier dysfunction, and microbiota-derived signals converge.

### 4.1. Synthesis and Degradation

The biosynthesis of CBG begins with the formation of CBGA, which is generated from olivetolic acid and geranyl pyrophosphate. CBGA serves as a central branching intermediate in the cannabinoid pathway and can be enzymatically converted into THCA, CBDA, or CBCA by specific oxidocyclase enzymes. Neutral cannabinoids, such as THC, CBD, CBC, and CBG, are subsequently generated mainly through the non-enzymatic decarboxylation of their acidic precursors, driven by heat, light exposure, or storage conditions [110].

Regarding its metabolism, CBG is rapidly metabolized by several human CYP450 isoforms, particularly CYP2J2, CYP3A4, CYP2D6, CYP2C8, and CYP2C9, generating oxidized metabolites such as cyclo-CBG and 6′,7′-epoxy-CBG. Some of these metabolites retain biological activity and exhibit anti-inflammatory properties. These findings suggest that hepatic CYP-mediated oxidation is a relevant metabolic pathway for CBG and that some metabolites may retain biological activity. Notably, CBG and its oxidized metabolites have been reported to reduce inflammatory responses in LPS-stimulated BV2 microglial cells, supporting the possibility that CBG metabolism may contribute not only to drug clearance but also to the generation of bioactive derivatives with anti-inflammatory potential [111]. 

In addition to phase I oxidation, recent pharmacokinetic evidence indicates that phase II metabolism also contributes to CBG disposition. In a pharmacokinetic study conducted in an equine model, CBG exhibited extensive distribution and high clearance, with CBG-glucuronide identified as a major circulating metabolite [112]. These findings support phase II glucuronidation as a relevant pathway in CBG biotransformation and disposition. Together, current evidence indicates that CBG disposition involves both CYP450-mediated oxidative metabolism and phase II conjugation, which may influence systemic exposure, bioavailability, metabolite formation, and potential pharmacological interactions.

### 4.2. Anti-Inflammatory and Analgesic Effect of Cannabigerol

Evidence of neuroinflammation supports CBG’s anti-inflammatory profile. In vitro studies have reported that CBG can reduce inflammatory and oxidative responses in cellular models of neuroinflammation, including the modulation of PPARγ- and nuclear factor erythroid 2-related factor 2 (Nrf2) ()-associated pathways, which are relevant to cellular antioxidant defense and inflammatory regulation [113]. In addition, the cannabigerol quinone derivative VCE-003 has been evaluated in a chronic Theiler’s murine encephalomyelitis virus-induced model of multiple sclerosis. In this model, VCE-003 improved motor function and attenuated neuroinflammatory responses in the brain and spinal cord. Mechanistically, this derivative activated PPARγ transcriptional activity, protected neuronal cells from excitotoxic damage, and reduced the release of pro-inflammatory mediators in LPS-stimulated microglial cells, supporting the relevance of the CBG scaffold in neuroimmune and demyelinating contexts [114].

Regarding analgesic activity, the evidence is more limited and should be interpreted cautiously. The molecular profile of CBG provides a plausible mechanistic basis for analgesic effects because it modulates TRPV1, TRPA1, and TRPM8 channels, as well as α2-adrenoceptor- and 5-hydroxytryptamine 1A receptor (5-HT_1A_-related signaling pathways involved in nociceptive processing, inflammatory hypersensitivity, descending pain modulation, and visceral sensitivity [106,107,115,116]. Moreover, synthetic CBG derivatives such as HUM-223, HUM-233, and HUM-234 have shown anti-inflammatory and antinociceptive properties in preclinical models.

The synthetic compounds HUM-223, HUM-233, and HUM-234 were evaluated in established in vivo models of inflammation and pain, including paw swelling/pain-related assays and a zymosan-induced arthritis model. HUM-223 reduced inflammatory activity in the affected joint by decreasing neutrophil elastase signaling and lowering expression of inflammatory genes such as neutrophil elastase (Elane), aggrecanase-1 (Adamts4), and myeloperoxidase (MPO). These findings suggest that synthetic CBG-derived compounds may attenuate inflammation by modulating neutrophil-associated inflammatory responses and tissue-degrading enzymes [117]. In addition, HUM-233 and HUM-234 also showed anti-inflammatory and analgesic effects, whereas HUM-234 displayed additional metabolic benefits in mice fed a high-fat diet, including reduced obesity-related alterations and hepatic steatosis [117].

Therefore, these findings support the pharmacological relevance of the CBG chemical scaffold for the development of anti-inflammatory and analgesic derivatives. However, as these compounds are synthetic derivatives and not native purified CBG, their effects cannot be directly extrapolated to CBG itself without comparative mechanistic and behavioral studies.

Although the molecular profile of CBG provides a plausible mechanistic basis for analgesic activity, direct behavioral evidence using purified CBG in classical pain models remains more limited than that available for THC or CBD. Nevertheless, recent preclinical studies have begun to support its antinociceptive potential, particularly in neuropathic pain conditions. Sepulveda et al. evaluated purified CBG in male and female C57BL/6 mice using three pain paradigms: cisplatin-induced peripheral neuropathy, the formalin test, and the tail-flick assay. CBG attenuated mechanical hypersensitivity in the cisplatin-induced neuropathy model, whereas its effects were less consistent in formalin-induced persistent pain and acute thermal nociception. These findings suggest that the analgesic profile of CBG may be more pertinent in chemotherapy-induced neuropathic pain than in acute inflammatory or thermal pain models. Although this study was mainly behavioral, the observed antinociceptive effect is consistent with the modulation of cannabinoid and non-cannabinoid targets involved in neuropathic pain signaling, including CB1, CB2, α2-adrenoceptors, and TRP channels [23].

Additional evidence supports the effects of repeated administration of isolated CBG on chemotherapy-induced neuropathic pain. Isolated CBG was evaluated in male and female mice using a cisplatin-induced peripheral neuropathy model. Repeated administration of isolated CBG significantly reduced mechanical hypersensitivity without evidence of tolerance or major adverse effects during the evaluated period [118].

In a recent study, a high-CBG *Cannabis sativa* extract reduced the acute and chronic hypernociception induced by formalin or spinal nerve ligation in rats exposed to prenatal hypoxia-ischemia. The molecular pathways involved were analyzed, showing reduced expression levels of TNF-α and voltage-gated sodium channel 1.7 (Nav1.7) in female and male rats [119].

Moreover, the same group of researchers evaluated an orally administered CBG-enriched extract in preclinical models of acute, inflammatory, and neuropathic pain. The extract showed antinociceptive effects and was associated with modulation of CB2 receptor signaling and reduced spinal dorsal horn microgliosis, suggesting a possible neuroimmune mechanism in nociceptive sensitization. However, because the formulation was a CBG-enriched extract rather than purified CBG, these findings support the analgesic potential of CBG-rich preparations but cannot be directly extrapolated to isolated CBG [120].

Overall, current evidence suggests that CBG may be particularly relevant in neuropathic pain, including chemotherapy-induced peripheral neuropathy, compared with acute pain models. However, additional studies using purified CBG, standardized formulations, dose–response curves, encompassing both sexes, employing multiple pain models, and employing clinically relevant routes of administration are required before establishing CBG as a therapeutic candidate for pain management.

Evidence from neuroinflammation-related models further supports the immunomodulatory potential of the CBG scaffold. An in vitro study showed anti-inflammatory and antioxidant effects of CBG associated with Nrf2 expression, suggesting activation of antioxidant defense mechanisms involved in neuroinflammation [113]. The anti-inflammatory potential of purified CBG has also been observed in peripheral and skin inflammation.

In a recent study, purified CBG (>99% purity) was evaluated in carrageenan-induced paw edema and in vitro inflammatory models, reporting decreased expression of inflammatory mediators such as COX-2, inducible nitric oxide synthase (iNOS), TNF-α, IL-1β, and IL-6, although the macroscopic reduction in paw edema was not statistically significant [121]. In a similar manner, CGB has demonstrated antinociceptive effects when administered intraperitoneally in the carrageenan-induced inflammatory pain, as well as the arthritis pain model and nerve pain model in mice. Pharmacological evidence from these preclinical models suggests that the antinociceptive effects of isolated CBG involve TRPV1 desensitization and CB2 receptor activation, with β-endorphin release proposed as a downstream component of this response [24]. Nevertheless, the relative contribution of each pathway to the overall antinociceptive effect of CBG requires further mechanistic characterization.

Similarly, the anti-inflammatory effects of CBG administered as an isolated compound were described in in vitro and in vivo models related to atopic dermatitis-like inflammation, associated with modulation of the of janus kinases (JAK)/signal transducer and activator of transcription proteins (STAT)/NF-κB signaling pathway. These findings suggest that CBG may regulate inflammatory signaling at the molecular level, even when tissue-level outcomes vary across models [122].

Emerging evidence also points to a possible role for CBG in immune-mediated joint inflammation. CBG formulated as a nanoemulsion was evaluated in a collagen-induced arthritis model in rats and demonstrated regulation of inflammatory pathways associated with arthritis progression. Although this line of evidence remains preliminary, it broadens the potential relevance of CBG beyond gastrointestinal inflammation and supports further evaluation in chronic immune-mediated inflammatory diseases [123].

Taken together, current findings suggest that CBG may act as a context-dependent anti-inflammatory modulator rather than as a selective cannabinoid receptor ligand. The strongest support remains preclinical and mechanistic, particularly in intestinal inflammation and neuroinflammation-related models. Nevertheless, translational conclusions remain limited by differences in formulation, dose, route of administration, and experimental design. Future studies should directly compare purified CBG with CBG-rich extracts and CBG-derived compounds, standardize experimental conditions, including both sexes, and integrate molecular markers with functional inflammatory outcomes.

### 4.3. Evidence in Irritable Bowel Syndrome and Gastrointestinal-like Inflammatory Models

Currently, direct evidence supporting the utilization of purified CBG in IBS is limited. Most available data originate from gastrointestinal inflammatory models, particularly inflammatory bowel disease (IBD)-like paradigms such as chemically induced colitis. Therefore, CBG should be discussed as a candidate compound for gastrointestinal inflammation, intestinal barrier dysfunction, and visceral pain-related conditions, rather than as an established therapeutic option for irritable bowel syndrome.

The rationale for exploring CBG in gastrointestinal disorders is supported by its interaction with pharmacological targets involved in intestinal homeostasis and visceral sensitivity, including cannabinoid receptors, TRP channels, α2-adrenoceptors, serotonergic signaling, and PPARγ-related pathways. These targets are relevant because gastrointestinal inflammation and visceral pain involve epithelial barrier disruption, immune activation, altered enteric signaling, microbial dysbiosis, and sensitization of peripheral and central nociceptive pathways. However, mechanistic plausibility should not be interpreted as evidence of clinical efficacy in IBS.

α2-adrenoceptors are expressed in intestinal epithelial cells and participate in the regulation of epithelial proliferation. Moreover, α2-adrenergic agonists such as clonidine have been investigated for their ability to reduce visceral sensitivity and colonic distension-related symptoms in IBS. Since CBG displays α2-adrenoceptor agonist activity, this receptor provides a plausible mechanistic link between CBG and visceral nociceptive modulation. However, a direct role for α2-adrenoceptor activation in CBG-mediated effects in experimental IBS has not yet been demonstrated [124,125].

In addition, the data indicate that the purified CBG reduced colon injury, nitric oxide production, and iNOS expression in a murine model of dinitrobenzene sulfonic acid-induced colitis [126]. These findings suggest that CBG may modulate intestinal inflammatory damage through mechanisms involving nitrosative stress and inflammatory mediator regulation.

More recent evidence has extended these observations to CBG-rich preparations. In a dextran sulfate sodium-induced model of colitis, treatment with a high-CBG hemp extract attenuated disease severity and was accompanied by a change in the polarization of gut microbial and metabolomic profiles [22]. However, because the preparation contained additional phytocannabinoids and other constituents, these microbiota-related effects cannot be attributed specifically to purified CBG.

Additional gastrointestinal evidence has emerged from studies using combined cannabinoid preparations [127], which reported that oral administration of CBD/CBG hemp extract reduced colitis severity and abdominal pain-like responses in a murine model of ulcerative colitis. This is relevant because abdominal pain and visceral hypersensitivity are important symptoms in both IBD and IBS. However, because the formulation contained both CBD and CBG, the effects cannot be exclusively attributed to CBG.

Overall, the current literature supports CBG and CBG-rich preparations as promising candidates for gastrointestinal inflammatory disorders and visceral pain-related conditions. However, evidence for IBS remains indirect and should be presented cautiously. Future studies should evaluate purified CBG in validated models of visceral hypersensitivity, intestinal motility, epithelial barrier dysfunction, and microbiota-dependent inflammation. In addition, controlled clinical studies are required to determine whether CBG has therapeutic relevance for IBS, IBD-associated abdominal pain or microbiota-associated gastrointestinal disorders.

### 4.4. Microbiota Modulator and Antimicrobial Properties of Cannabigerol

Particularly, CBG has been evaluated as purified CBG, CBG-rich hemp extracts, and other CBG-containing preparations in preclinical in vivo and in vitro models [121,128,129,130], showing antioxidant, anti-inflammatory, and antinociceptive effects. Likewise, microbiota-associated effects and antimicrobial activity have also been explored.

In this regard, the modulating microbial character of cannabinoids has been demonstrated by promoting intestinal symbiotic bacteria and suppressing certain pathogenic bacteria, thereby restoring host homeostasis and establishing a mechanism to mitigate inflammation, structural damage, and associated symptoms. The treatment with high-CBG hemp extract was associated with changes in gut microbiota composition and improved inflammatory outcomes in an experimental model of colitis [22]. However, because this preparation contained additional phytochemicals, these microbiota-related effects cannot be attributed exclusively to purified CBG. In parallel, purified CBG has demonstrated direct antimicrobial activity against selected bacterial pathogens, including effects associated with bacterial membrane disruption and inhibition of biofilm formation [131]. Taken together, these findings support microbiota-associated effects of CBG-rich preparations and direct antimicrobial activity of purified CBG against selected bacterial species.

## 5. How Do Cannabigerol and the Gut Microbiota Interact to Modulate Inflammation and Pain Responses?

**Figure 4:**
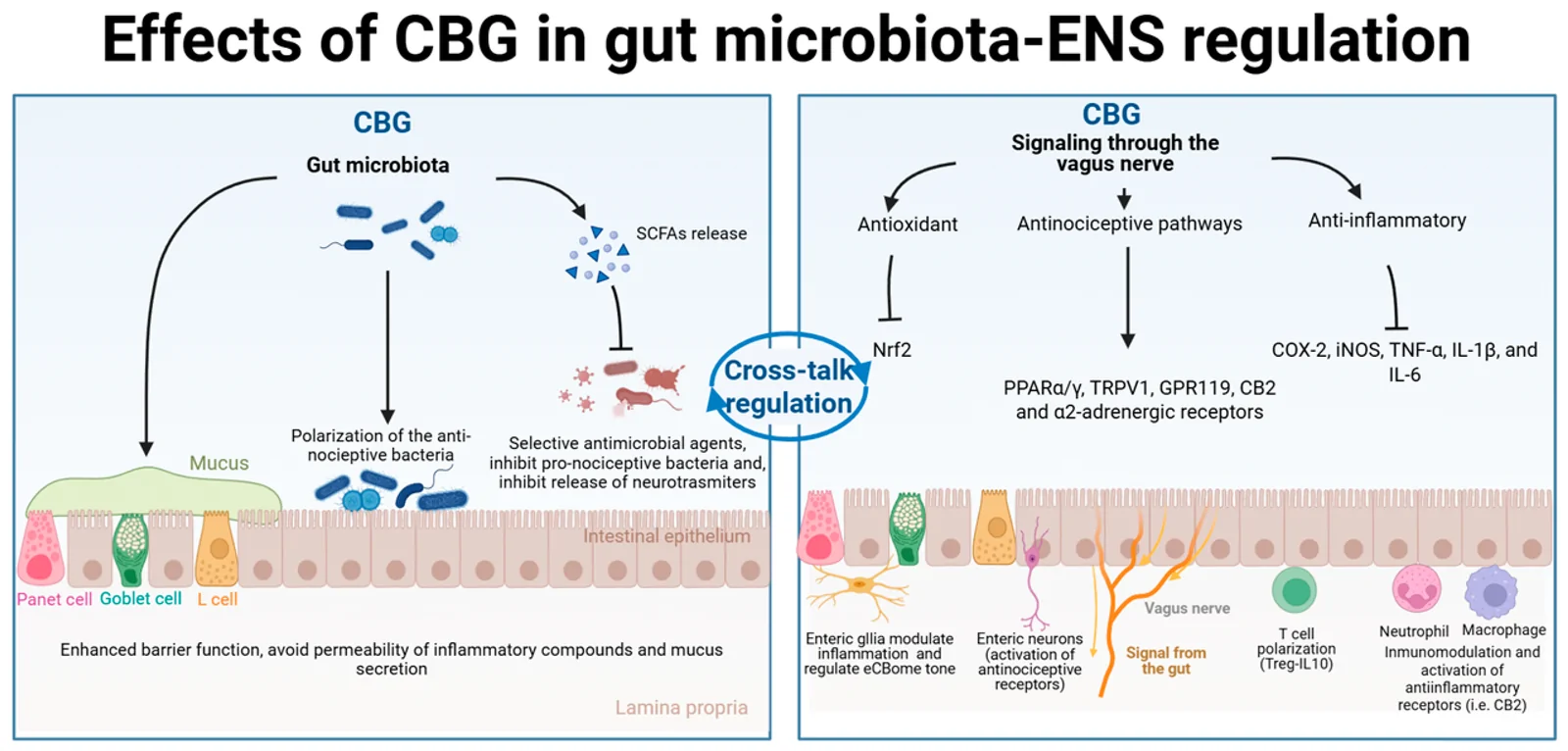
Proposed conceptual model of the potential interactions among cannabigerol (CBG), the gut microbiota, and the enteric nervous system (ENS). The diagram summarizes mechanisms suggested by available preclinical evidence related to inflammatory and nociceptive regulation. In the left panel, CBG-rich preparations have been associated with changes in gut microbial composition and metabolomic profiles, including pathways related to short-chain fatty acids (SCFAs), which may contribute to intestinal barrier integrity and immune regulation. However, these microbiota-related effects cannot be attributed specifically to purified CBG. In the right panel, CBG is represented in relation to molecular targets involved in inflammatory and nociceptive signaling, including cannabinoid receptors, TRP channels, α2-adrenoceptors, and peroxisome proliferator-activated receptor (PPAR)- related pathways. CBG has also been associated with changes in inflammatory mediators such as cyclooxygenase-2 (COX-2), inducible nitric oxide synthase (iNOS), tumor necrosis factor-alpha (TNF-α), interleukin 1 beta (IL-1β), and interleukin 6 (IL-6)6 in preclinical models. Enteric neurons, immune cells, epithelial cells, and enteric glial cells participate in these signaling pathways and may contribute to communication with the central nervous system through neural and neuroimmune mechanisms. Overall, the figure represents a proposed functional interface based on available evidence rather than an established causal CBG effect on the gut microbiota–eCBome pathway. Arrows indicate activation effects, blunt arrows indicate inhibitory effects, and circular arrows represent the cross-talk regulation between gut microbiota and eCBome on ENS.

The interplay of CBG with the gut microbiota-eCBome axis establishes signaling pathways that potentially modulate pain, inflammation, and other pathological disorders. Available preclinical evidence suggests a potential functional interplay among CBG, gut microbiota, and eCBome signaling. CBG may influence molecular targets associated with the eCBome [22], while CBG-rich preparations have been associated with changes in gut microbial composition [22]. These observations provide a mechanistic basis for proposing the gut microbiota–eCBome interface of CBG in the modulation of inflammatory and nociceptive processes. However, direct experimental evidence demonstrating causal and bidirectional interaction between these three components remains limited. The microbiota comprises the gut bacterial community involved in metabolizing fiber to produce SCFAs and contributing to the maintenance of the protective barrier of the intestine [29]. In turn, the eCBome represents an internal network of receptors, ligands, and enzymes, which controls how to respond to pain and inflammatory signals [54]. In parallel, other inflammatory targets are activated as part of the pleiotropic or indirect effects of receptor activation and modulation (Figure 4).

These pathways may converge on biological processes relevant to inflammation and nociception, including maintenance of intestinal barrier integrity, modulation of microbial metabolite production, regulation of neuroimmune signaling, and changes in immune cell activation. Despite the current evidence, it has not been established that CBG directly regulates the gut microbiota and eCBome-dependent processes; the existence of a causal mechanism linking these three elements remains to be experimentally demonstrated.

### 5.1. Gut Microbiota Biotransformation of CBG

Evidence regarding direct microbial metabolism of CBG remains limited. The gut microbiota can directly transform CBG or use enzymes such as ß-glucuronidase, which plays a role in glucuronic deconjugation of THC metabolites, cycling back into circulation as more potent anti-inflammatory metabolites (7-hydroxy-CBD and 11-hydroxy-THC) than their precursors or conjugated metabolites [78,132,133]. The relationship between ß-glucuronidase and CBG has not yet been reported.

Therefore, it is more accurate to propose that the gut microbiome may influence CBG pharmacokinetics through intestinal absorption, bile acid metabolism, epithelial barrier integrity, enterohepatic cycling, and microbial β-glucuronidase activity, as described for cannabis family compounds. The gut microbial transformation of CBG into antinociceptive or anti-inflammatory metabolites warrants further exploration and research.

However, recent preclinical evidence indicates that high-CBG hemp extract can modulate gut microbial composition in experimental colitis, favoring the anti-nociceptive bacteria and suppressing some pro-nociceptive bacteria [22]. This supports the relevance of CBG as an external stimulus on the gut microbiota-eCBome axis in gastrointestinal inflammation. Up to this point, CBG seems to function as a potential microbiota modulator, positively preserving symbiotic bacteria and selectively inhibiting pathobionts.

### 5.2. CBG Interplay with Gut Microbiota-eCBome Axis

Growing evidence supports bidirectional interactions between the eCBome and the gut microbiota in the regulation of inflammatory and nociceptive processes. However, the specific contribution of CBG to this interface remains insufficiently characterized and represents an emerging area of investigation. Evidence derived from endogenous cannabinoids and other cannabinoid-related mediators provides mechanistic context but should not be interpreted as direct evidence of CBG-mediated microbiota–eCBome regulation. Nociceptive receptors and sensory signaling pathways are widely distributed throughout the gastrointestinal tract, enteric nervous system, vagal afferents, and spinal sensory pathways. These pathways can be activated or modulated by SCFAs, peptidoglycans, polyunsaturated fatty acids (PUFAs), pH changes, proteases, formyl peptides, nitrites, nitric oxide, reactive oxygen species (ROS), and by the production of neurotransmitters and hormones, which occur under physiological conditions and during microbiota dysfunction. These mediators may induce pleiotropic responses in immune, epithelial, and secretory cells. Following nociceptor activation, nociceptive signals are transmitted through vagal, sympathetic, parasympathetic, and spinal afferent pathways to central and supraspinal regions involved in pain processing. In addition, some products associated with gut microbiota dysfunction may enter the systemic circulation and influence spinal cord and brain structures involved in nociceptive processing [45].

Evidence from studies using isolated CBG supports interactions with several molecular targets relevant to nociceptive and inflammatory signaling, including cannabinoid receptors, α2-adrenoceptors, and TRP channels [23,54,95,106,107,115,117,119,122,134]. Additionally, cannabinoid-containing preparations have provided relevant information to understand the relationship between CBG and these signaling pathways [23,54,95,106,107,115,117,119,134].

Other components of the eCBome, including GPR119 and related signaling pathways, may contribute to gut microbiota communication; however, these should not be considered established, direct molecular targets of CBG unless specifically demonstrated experimentally [23,54,95,106,107,115,117,119].

One of the most impactful findings to understand how microbiota regulates the eCBome tone in the gut, driving antinociceptive response, was proposed by Rousseaux et al. [135]. Evidence from other endocannabinoidome-related mediators provides additional mechanistic context. For example, the administration of OEA, an endogenous PPARα agonist, has been associated with changes in gut microbial composition and reductions in inflammatory mediators in mice [136]. Although these findings illustrate potential interactions between eCBome-related signaling and the gut microbiota, they cannot be directly extrapolated to CBG.

Additional information supporting gut microbiota–eCBome interactions comes from studies of *Lactobacillus acidophilus* NCFM. The oral administration of this strain increased the expression of μ-opioid and CB2 receptors in colonic epithelial cells and produced dose-dependent reductions in intestinal pain models [135]. These findings indicate that microbial signals can influence cannabinoid receptor-dependent visceral nociception; however, they do not demonstrate that CBG participates directly in this microbiota–CB2 interaction.

Given that the gut microbiota and eCBome can be modulated by diet, exercise, and physiological conditions, their pleiotropic functions were demonstrated in a six-week exercise intervention study, in which levels of AEA, OEA, and PEA were increased, but not in the control groups. In the same study, increased endocannabinoid levels were associated with gut microbiota diversity and increased SCFAs produced by bacteria such as *Bifidobacterium*, *Coprococcus*, and *Faecalibacterium*, contrasting with a negative association with *Collinsella*. Increased endocannabinoids such as AEA and PEA were related to increased butyrate production and decreased levels of the inflammatory cytokines TNF-α and IL-6. In the same study, AEA and OEA correlated with the CB2 receptor and SCFAs, and free fatty acid receptor 2 (FFAR2). Data indicate that butyrate produced by the gut microbiota has an anti-inflammatory effect, which is partially mediated by increased endocannabinoid tone induced by exercise, proposing the existence of other pathways in the modulation of inflammatory response through the gut microbiota; hence, its effects could be extended to modulate pain conditions [137].

Specifically, the biosynthesis of butyrate promotes CB2 receptor signaling in immune cells through the transition of pro-inflammatory to anti-inflammatory macrophages and the reduction in pro-inflammatory cytokines in an experimental colitis mouse model [138]. These mechanisms may be related to the control of intestinal pain. Since CBG targets the TRPV1 receptors, which in turn stimulate goblet cells for mucus secretion in the gut, the ablation of receptors produces dysbiosis characterized by a reduction in the levels of bacterial *Lactobacillus* spp. and *Clostridia* spp., as well as their metabolites acetate and butyrate, affecting their abundance [139].

THC and CBD have demonstrated amelioration in experimental autoimmune encephalomyelitis (EAE), a murine model of Multiple Sclerosis (MS), through reduction in the inflammatory response. Several mechanisms of action are involved, including reducing interleukin 17 (IL-17) and IFN-γ production, increasing IL-10, decreasing immune cell infiltration in the brain, and preventing LPS release associated with Gram-negative bacteria and the accumulation of mucin-degrading bacteria, which can lead to gut microbial dysbiosis. Likewise, the production of SCFAs, such as butyric, isovaleric, and valeric acids, was another mechanism proposed to decrease inflammation and gut dysbiosis in AEA mice [140,141]. Moreover, the administration of PEA induces modifications in gut microbiota and changes in spinal levels of cannabinoids and receptors in vitamin D deficiency-induced neuropathic pain, highlighting the restoration of 2-AG and CB1 receptor in the gut and spinal cord, respectively [142].

In this regard, oral administration of a CB1 antagonist increased *Akkermansia muciniphila*, which in turn regulates the permeability and secretion of gut peptides, as well as the tone of endocannabinoids such as 2-OG, 2-AG, and 2-PG in the intestine [143,144]. 2-AG is a potent anti-inflammatory and anti-pain mediator. Similarly, 2-PG and 2-OG exert antinociceptive effects by desensitizing other endocannabinoid receptors, such as TRPV1 [145,146,147]. CBG supplementation for two weeks has also been reported to protect against inflammation in colon tissue. This is attributed to reduced arachidonic acid in colon tissue, which subsequently limits the activity of COX1, COX2, cytosolic phospholipase A2 (cPLA_2_), and LOX enzymes and promotes lipoxin A production. This phenomenon is observed even under high lipid and sugar stress conditions [130].

These findings support an interaction between CBG and lipid signaling pathways associated with inflammatory regulation; however, the extent to which these effects involve the eCBome or depend on the gut microbiota remains unknown. Taken together, the available evidence supports a biologically plausible interface among CBG, gut microbial processes, and eCBome signaling. Nevertheless, most mechanistic links are currently inferred from studies involving endogenous cannabinoids, other phytocannabinoids, microbial metabolites, CBG-rich preparations, or other CBG-containing formulations, rather than from experiments specifically designed to demonstrate a causal CBG effect on the gut microbiota–eCBome pathway. Therefore, direct mechanistic studies using purified CBG, microbiota-controlled experimental models, standardized formulations, and validated inflammatory and nociceptive outcomes are required before this interaction can be considered an established therapeutic mechanism.

## 6. Conclusions

CBG is a promising broad-spectrum agent due to its pharmacological characteristics that can affect signaling pathways and modulate gut microbiota. Further exploration is required to understand the metabolites, receptors, and signaling pathways that couple the gut microbiota with the host eCBome and the immune and pain pathways. Specifically, a preclinical detailed analysis of the relationship between CBG and gut microbiota and its mechanism of action to alleviate pain and inflammation is required. Moreover, the prospects of CBG include conducting controlled clinical studies to elucidate its pharmacokinetics, pharmacodynamics, toxicology, pharmaceutical technology, and safety profile. Notably, therapies based on the interaction between CBG and the modulation of gut microbiota to alleviate inflammation and pain hold significant potential for research.

In this context, and based on the reviewed literature, we propose that CBG could act by direct activation of the α -adrenergic receptor, which may constitute a newly identified molecular target to improve mucosal healing, intestinal inflammation, and wound healing. In support of this, the stimulation of this receptor with a specific α-receptor agonist, like CBG, produces antinociception and could promote epithelial proliferation and stem cell function. Simultaneously, it reduces differentiation and causes positive changes in the composition of gut microbiota, preserving symbiotic bacteria and selectively controlling proinflammatory bacteria.

Nevertheless, based on evidence, other proposed targets include CB2 and PPARα/γ, which exert potent anti-inflammatory and analgesic effects in the ENS, as well as TRPV1 desensitization involved in the pain and motility process. Likewise, a direct effect of CBG could be mediated by suppression of inflammatory enzymes and cytokines such as TNFα, COX-2, iNOS, IL-1β, IL-6, and by increasing antioxidant factors such as Nrf2. Finally, the effects of CBG could be mediated through the potential modulation of the gut microbiota, establishing a bidirectional relationship between the gut microbiota and eCBome.

## Data Availability

No new data were created or analyzed in this study. Data sharing is not applicable to this article.

## Abbreviations

The following abbreviations are used in this manuscript:

2-AG	2-arachidonoylglycerol
2-AGE	2-arachidonyl glyceryl ether
2-LG	2-linoleoyl-glycerol
2-OG	2-oleoyglycerol
2-PG	2-palmitoyglycerol
5-HT	5-hydroxy tryptamine
ABHD	αβ hydrolase 6
ABHD12	αβ hydrolase
Adamts4	Aggrecanase-1
AEA	Anandamide
cAMP	Cyclic adenosine monophosphate
CB1	Cannabinoid receptor type 1
CB2	Cannabinoid receptor type 2
CBCA	Cannabichromenic acid
CBD	Cannabidiol
CBDA	Cannabidiolic acid
CBG	Cannabigerol
CNS	Central nervous system
COX-1	Cyclooxygenase-1
COX-2	Cyclooxygenase-2
cPLA_2_	Cytosolic phospholipase A2
CYP450	Cytochrome P450
DAG	Diacylglycerol
DAGL	Diacylglycerol lipase
DHEA	*N*-docosahexaenoyl-ethanolamine
DRG	Dorsal root ganglia
EAE	Encephalomyelitis
eCBome	Endocannabinoidome
EGCs	Enteric glial cells
Elane	Neutrophil elastase
ENS	Enteric nervous system
EPOX	Epoxygenase
FAAH	Fatty acid amide hydrolase
FFAR2	Free fatty acid receptor 2
GABA	Gamma-aminobutyric acid
Gαi	Inhibitory G protein alpha subunit
Gαq	Gq protein alpha subunit
Gαs	Stimulatory G protein alpha subunit
GPR119	G protein-coupled receptor 119
GPR55	G protein-coupled receptor 55
IBD	Inflammatory bowel disease
IBS	Irritable bowel syndrome
IL-1β	Interleukin 1 beta
IL-6	Interleukin 6
IL-10	Interleukin 10
LEA	*N*-linoleoyl-ethanolamine
LOX	Lipoxygenase
MAGL	Monoacylglycerol lipase
MAPK	mitogen-activated protein kinase
MPO	Myeloperoxidase
MS	Multiple Sclerosis
NAAA	*N*-acylethanolamine acid amidase
NADA	*N*-arachidonoyl dopamine
NAPE	*N*-acylphosphatidylethanolamide
Nav1.7	Voltage-gated sodium channel 1.7
NAPE-PLD	*N*-acylphosphatidylethanolamine phospholipase
NF-kB	Nuclear factor kappa B
Nrf2	Nuclear factor erythroid 2-related factor 2
NSAIDs	Non-steroidal anti-inflammatory drugs
OEA	Oleoylethanolamide
PEA	*N*-Palmitoylenthanolamide
PNS	Peripheral nervous system
PPARα	Peroxisome proliferator-activated receptor α
PPARγ	Peroxisome proliferator-activated receptor γ
PUFAs	Polyunsaturated fatty acids
RegIIIβ	Regenerating islet-derived protein 3 beta
RegIIIγ	Regenerating islet-derived protein 3 gamma
ROS	Reactive oxygen species
SCFAs	Short-chain fatty acids
SEA	*N*-Stearoylethanolamide
STAT	Signal transducer and activator of transcription proteins
TLR2	Toll-like receptor 2
TLR4	Toll-like receptor 4
TG	Trigeminal ganglia neurons
Th1/Th17	T helper cells
THC	Δ9-tetrahydrocannabinol
THCA	Tetrahydrocannabinolic acid
TNF-α	Tumor necrosis factor-alpha
Tregs	Regulatory T lymphocytes
TRPV1	Transient receptor potential cation channel subfamily V member 1
virodhamine	O-arachidonoyl-ethanolamine
